# PREVALENCE OF CONGENITAL ANOMALIES AND THEIR ASSOCIATED FACTORS IN NEWBORNS IN THE CITY OF SÃO PAULO FROM 2010 TO 2014

**DOI:** 10.1590/1984-0462/;2017;35;1;00002

**Published:** 2017

**Authors:** Henrique Willian Cosme, Laura Silva Lima, Lene Garcia Barbosa

**Affiliations:** a Universidade Anhembi Morumbi, São Paulo, SP, Brasil.

**Keywords:** Congenital anomalies, Prevalence, Association, Causes, Newborns, São Paulo City

## Abstract

**Objective:**

To study the prevalence of congenital anomalies in newborns in the city of São Paulo from 2010 to 2014, as well as to analyze other variables associated with the anomalies.

**Methods:**

Data was collected from the Ministry of Health’s Live Births Information System (SINASC) from 2010 to 2014 in São Paulo City. The variables analyzed were length and type of pregnancy, maternal age, and ethnicity and sex of the newborn. The absolute and relative frequencies of congenital anomalies were verified, and the variables associated with them were calculated with the *odds ratio* (OR) and a 95% confidence interval.

**Results:**

A total of 819,018 live births occurred in the city of São Paulo, and in 14,657 (1.6%) of them, some congenital anomaly was reported. The most frequent congenital anomalies found were those related to osteoarticular system followed by those related to the cardiovascular system. Risks associated with the presence of congenital anomalies were observed in the following factors: maternal age over 40 years (OR=1.59; 95%CI 1.47-1.71), multiple pregnancies (OR=1.28; 95%CI 1.19-3.77), and low birth weight (OR=3.35; 95%CI 3.21-3.49). The female gender was considered a protective variable (OR=0.78; 95%CI 0.75-0.81).

**Conclusions:**

Congenital anomalies are responsible for morbidity and mortality in the neonatal period. Their early diagnosis is important for planning and resource allocation of specialized health services directed toward the families and infants.

## RESUMO

**Objetivo:**

Estudar a prevalência de anomalias congênitas em nascidos em maternidades do município de São Paulo, no período de 2010 a 2014, assim como analisar possíveis fatores associados às anomalias.

**Métodos:**

Coleta de dados do Sistema de Informações sobre Nascidos Vivos (Sinasc) do Ministério da Saúde, no período de 2010 a 2014, no município de São Paulo. As variáveis analisadas foram: duração e tipo de gestação, idade materna, etnia e sexo do recém-nascido. Foram verificadas as frequências absoluta e relativa de anomalias congênitas, e realizou-se o cálculo do *Odds Ratio* (OR) e de seu intervalo de confiança.

**Resultados:**

Foram notificados 819.018 nascidos vivos no município de São Paulo no período, dos quais 14.657 (1,6%) tiveram algum tipo de anomalia congênita. As malformações congênitas mais comuns encontradas foram, em primeiro lugar, as osteoarticulares e, depois, as do sistema circulatório. Foi observada associação de anomalias congênitas com idade materna superior a 40 anos (OR=1,59; IC95% 1,47-1,71), gestações múltiplas (OR=1,28; IC95% 1,19-3,77), recém-nascidos com baixo peso (OR=3,35; IC95% 3,21-3,49), sendo o sexo feminino fator de proteção (OR=0,78; IC95% 0,75-0,81).

**Conclusões:**

As anomalias congênitas são causa de morbimortalidade no período neonatal, e o seu diagnóstico precoce é para o planejamento e a alocação de recursos dos serviços de saúde especializados.

## INTRODUCTION

Congenital anomalies are developmental disorders of embryonic origin. They are present at birth, include a high morbidity, and represent one of the main causes of infant mortality. Their etiology is associated with physical, chemical, biological, or genetic environmental factors.^1^

Close to 60% of congenital anomalies have an unknown origin. Genetic congenital anomalies, such as chromosomal abnormalities, have a fair amount of research done on them while those of environmental etiology, caused by teratogens, are the least investigated.^2^Among the causes of congenital anomalies, infectious agents, environmental agents like radiation, mechanical factors, and chemical compounds in addition to maternal diseases stand out.^3^Some maternal factors like age, lifestyle, type of pregnancy, maternal health, among others, have been researched and connected to the occurrence of congenital anomalies.^4^

The Health Ministry in 1990, initiated the Live Births Information System (Sinasc), with the goal of gathering information relative to the births that have taken place in all national territory, which allows for the completion of more detailed epidemiological studies. In 1999, Sinasc was instituted as a new field, and named field 34, which gave the opportunity to record congenital anomalies. When it is filled out, the record allows the research of the anomalies’ frequency and the nature of their occurrence in order to create reliable health and surveillance indicators, which will ultimately make health policy planning, specifically child health policy planning, easier. In the city of São Paulo, this system was implanted in 2000, with the purpose of registering all of the live births in the municipality.^5^

The objective of this study was to investigate the prevalence of congenital anomalies in births in maternities in São Paulo in the period from 2010 to 2014, using the database from the Health Ministry (Sinasc), in order to analyze possible factors associated with congenital anomalies.

## METHOD

This is a cross-sectional study, in which the database of the national record, the Sinasc from the Health Ministry,^6^was used in the period from 2010 to 2014, in the municipality of São Paulo. The following data was collected: length and type of pregnancy, maternal age, and ethnicity and sex of the newborn, birth weight, and type of congenital anomaly. 

The variables analyzed were:

- Length of pregnancy, divided into three categories: preterm: less than 37 weeks; term: 37 weeks to 41 weeks and 6 days; and post-term: over 42 weeks.
- Maternal age, divided into three categories: less than 19 years old, between 19 and 40 years old, and over 40 years old.
- Weight at birth, divided into three categories: between 500 and 2500g, between 2500g and 3550g, and over 3550g.
- Ethnicity, divided into five categories: white, black, yellow, brown, and indigenous.
- Type of pregnancy, divided into three categories: single pregnancy, twins, triplets, or more.
- Sex, divided into the categories of male and female.

Pregnancies with a duration of less than 22 weeks and newborns with a birth weight of less than 500g were excluded.

Congenital anomalies are registered in the Sinasc according to the International Classification of Sicknesses and Health Problems (CID) 10^7^and were grouped into ten categories based on CID 10 for better analysis of the data: chromosomopathies (Q90-99); abnormalities of the nervous system (Q00-07); head and neck abnormalities (Q10-18 and Q30-38); respiratory system abnormalities (Q32 and Q33); cardiovascular anomalies (Q20-28); anomalies of the digestive system (Q39-45); renal anomalies (Q60-64); osteoarticular anomalies (Q65-79); genital abnormalities (Q50-56); and others such as skin, lymphatic anomalies etc.

The absolute and relative frequencies were verified and an *odds ratio* was estabilished. An evaluation of the statistical association was tested by the chi-square test in the statistical program Stata 13.1 (Stata Corp, Texas, United States) and by the EZR software version 1.27, available on the website http://www.jichi.ac.jp/saitama-sct/SaitamaHP.files/statmed.html. The 95% confidence interval (95%CI) was reported in order to obtain health indicators and health surveillance indicators (prevention).

This study was approved by the Committee of Ethics in Research from the Anhembi Morumbi University, through the Brazil Presentation Platform Certificate for Ethics Assessment (CAAE) n. º 41629415.9.0000.5492.

## RESULTS

From 2010 to 2014, 819,018 live births were recorded in the city of São Paulo, of which 14,657 had some type of congenital anomaly, corresponding to a prevalence of 17.9 cases for every 1,000 live births throughout the time period studied (Table 1).

**Table 1:**
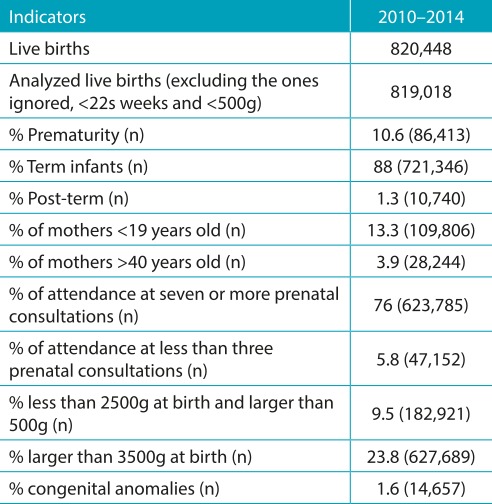
Health indicators in the city of São Paulo in the period from 2010 to 2014.

Source: http://www.prefeitura.sp.gov.br/cidade/secretarias/saude/tabnet/nascidos_vivos/index.php?p=159923. Live births, number of live births from mothers, who are residents and who gave birth in the municipality of São Paulo; prematurity, greater than 22 weeks and less than 37 weeks of pregnancy.

In Table 2, absolute and relative frequencies of the anomalies described in the period are observed, in accordance with the effected system. The majority (30% of the recorded cases) were malformations in the osteoarticular system, 25% in the circulatory system, and 13% in the head and neck.

**Table 2:Types:**
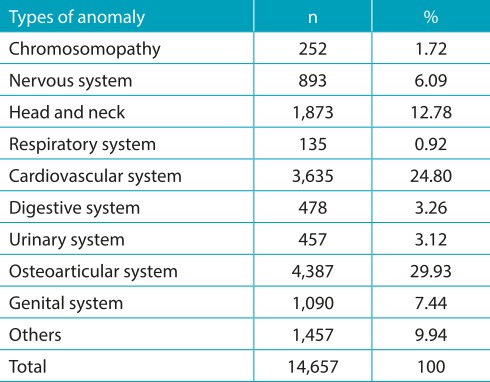
of anomalies in newborns with congenital anomalies in the period from 2010 to 2014, São Paulo.

Upon analyzing the factors that could be associated with congenital anomalies, a greater chance of anomalies in premature babies (OR=2.39; 95%CI 2.30-2.49) was found; in pregnant women older than 40 years old (OR=1.59; 95%CI 1.47-1.71) and women younger than 19 years old (OR=1.12; 95%CI 1.07-1.17); in newborns with a birth weight between 500 and 2500g (OR=3.35; 95%IC 3.21-3.49), and with a weight greater than 3550g (OR=1.52; 95%CI 1.46-1.58); and in multiple-baby pregnancies (triplets or more: OR=2.68; 95%CI 1.91-3.77; twins: OR=1.28; 95%CI 1.19-3.77). With respect to ethnicity, there was a higher prevalence of congenital anomalies in black and yellow women, OR of 1.35 and 1.24, respectively. With respect to sex, there was less prevalence in the female sex (OR=0.78; 95%CI 0.75-0.81) (Table 3).

**Table 3:**
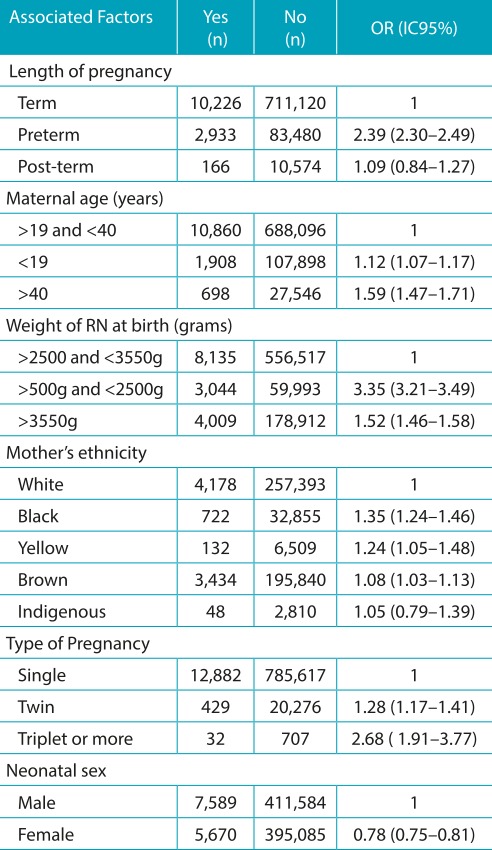
Factors associated with congenital anomalies and newborns, in the period from 2010 to 2014.

OR: *odds ratio*; IC95%: 95% confidence interval.

With respect to the most prevalent congenital anomalies, in accordance with the ten categories that were allocated, the following were obtained: chromosomal disorders (Down syndrome was the most prevalent, with 70% of chromosomal abnormalities); nervous system abnormalities, of which hydrocephalus (27%) and spina bifida (25%) were the most prevalent; respiratory system anomalies, especially lung malformations (85% of respiratory abnormalities); cardiovascular changes, with septal defects amounting to 41% of cardiac malformations; digestive system abnormalities, the most common being esophageal malformations (27%) and atresia or stenosis cervical abnormalities (22%); malformations of the urinary system, with 42% of renal origin, and 34% ureteral; osteoarticular (polydactyl, 32%, and congenital deformities of the feet, 27%), head and neck malformations (cleft palate, 21%, and other tongue and pharynx defects, 29%); and genital abnormalities, with a higher prevalence of hypospadias (47%) and cryptorchidism (35%).

## DISCUSSION

The prevalence of congenital defects in live births of the present study was 1.6 for every 100 live births, a lower incidence than described by Marques-de-Faria et al. in 2004 in the Brazilian population (1.4-5%), and greater than what was found by Bonifácio et al. in 2011^8^ ^,^ ^9^, which can reveal the underreporting of congenital reporting in the field 34 in the live births declaration, even after the detailed improvement efforts made by Luquetti and Koifaman in 2010. ^10^

On an average three million births per year occur in Brazil, of which approximately 60 thousand have congenital anomalies. The characterization of these congenital anomalies is important information for the planning and implementation of programs that take care of patients with these conditions, and their families. To better record data in the Sinasc, a partnership between the Health Ministry and the Medical Genetics Center of the Federal University of São Paulo was formed in 2005, and in 2008 a handbook was created in order to reduce the amount under-reporting and encourage early diagnosis of congenital defects. They also allowed each municipality to build their own clinical and epidemiological reference to reach a certain standard of excellence.^11^

During the study period, there was a predominance of infants with malformations of osteoarticular apparatus, especially polydactyly and foot deformities (a total of 59.2%), followed by malformations in the cardiovascular system and the head and neck, findings that resemble those found in other national studies and in first world countries like the United States and Europe. ^12^ ^,^ ^13^ ^,^ ^14^ The predominance of ostearticular malformations could be related to the easiness with which it is diagnosed. Osteacrticular malformations are visible during the physical examination at the moment of birth.

A greater statistical risk of congenital anomalies was identified in premature babies than in those born after 37 or more weeks of pregnancy, data that was also observed in the state of Rio de Janeiro^13^A greater chance of congenital anomalies was found in multiple pregnancies and, according to some authors, having twins is an important cause of congenital defects. The largest number of cases of congenital anomalies in multiple pregnancies can be explained, in part, by errors in cell divisions (genetic factors) and by environmental factors in utero, constricting of the amniotic band or umbilical cord, for example.^14^ ^,^ ^15^ ^,^ ^16^ ^,^ ^17^ Multiple pregnancies are associated with a larger number of premature births, and congenital anomalies can lead to premature delivery, implying high morbidity and mortality rates.

The extreme high and low ages are related to a higher number of perinatal complications. In our study, a high number of neonates with congenital anomalies, particularly chromosomal abnormalities, were verified coming from women on both ends of the age spectrum. Illnesses such as diabetes and hypertension tend to appear with greater frequency in older individuals. Thus, pregnancies in older women have a higher incidence of diabetes, arterial hypertension, and consequently, a high probability of perinatal complications, like abortion, congenital anomalies, preeclampsia, eclampsia, and premature births, among others. In order to better accompany these pregnancies, which are considered high risk, there is the necessity for specialized health services and the performance of pre-natal examinations to do a morphological evaluation, genetic studies, in the follow up of these pregnant women at risk.^18^ ^,^ ^19^ ^,^ ^20^

Among chromosomal abnormalities, the most frequent was Down syndrome (70% of cases). This finding was similar to the literature, both in relation to the total chromosomal abnormalities as well as the type of chromosomal abnormalities. Advanced maternal age is one of the factors involved in this result because it is associated with the higher incidence of aneuploidy. ^18^ ^,^ ^19^ ^,^ ^20^

Hydrocephalus and spina bifida were the most frequent congenital anomalies of the central nervous system (data similar to that found in the literature), and hydrocephalus may be associated with spina bifida and myelomeningocele (neural tube defects). Genetic factors, weight of the newborn at birth (low weight), length of pregnancy (prematurity), maternal age (both ends of the extreme), and the deficiency of maternal folic acid intake may be linked to neural tube defects.^21^ ^,^ ^22^ ^,^ ^23^ ^,^ ^24^ ^,^ ^25^ ^,^ ^26^ ^,^ ^27^

The malformations of the urinary and genital system are intimately inter-related because of embryological development and are generally more frequent in males.^28^According to De Paula and Gerra Júnior, because of the greater complexity of internal and external genital formation in males, there is a higher incidence of malformations in the male urogenital system, not to mention the tendency toward a higher number of documentation of malformations in this sex in cases of genital ambiguity. Besides this, the authors verified the association of genital ambiguity with malformations of the osteoarticular, cardiovascular, and digestive systems, as well as low birth weight and prematurity.^29^In general, males have a higher chance of congenital anomalies, a result that is similar to what was found in the literature.^14^ ^,^ ^25^ ^,^ ^26^

Congenital anomalies are associated with infant morbidity and mortality, especially in the neonatal period, which makes their early diagnosis important for the planning and allocation of specialized health service resources (prenatal, natal and post-natal), for the reduction of morbidity and mortality, especially in the early neonatal period, and for the improvement of quality of life and survival rates.

Because of the limitations of this study, it was difficult to identify the cases of multiple anomalies. They are an important cause of death during the neonatal period.^30^ ^,^ ^31^ ^,^ ^32^ Because the database Sinasc was used, it was not possible to link each case with the maternal data (age, drug use, number of pre-natal consults, education, and number of pregnancies) and neonatal data (gestational age, weight, and sex) as described in other studies that gathered information directly from medical records. As such, there is a need of further research on the subject.^26^
